# Influence of *Achyranthes japonica* extracts supplementation in the diets of finishing pigs

**DOI:** 10.1002/vms3.535

**Published:** 2021-05-26

**Authors:** Xiao Liu, In Ho Kim

**Affiliations:** ^1^ Department of Animal Resource and Science Dankook University Cheonan‐si Chungnam Korea

**Keywords:** *Achyranthes japonica* extracts, blood characteristics, growth performance, nutrient digestibility, pigs

## Abstract

**Background:**

Herbs and their extracts have been used for a long time in animal industries as alternatives to an antibiotic.

**Objectives:**

This study was evaluated the effects of dietary supplementation of *Achyranthes japonica* extracts (AJE) on the performance and production parameters in finishing pigs.

**Methods:**

Totally, 100 pigs with an average body weight of 50.33 ± 4.61 kg were used as a 10‐week feeding trial. Pigs (five replicates, three barrows and two gilts per pen) were allotted randomly to four treatments as the addition of 0%, 0.05%, 0.10% and 0.20% of AJE in basal diets.

**Results:**

A linear increase (*p* < .05) in average daily gain was observed during week 5, week 10 and overall period, and a linear decrease (*p* < .05) was observed in the feed conversion ratio during week 5. A linear increase (*p* < .05) in dry matter, protein digestibility and faecal ammonia emission on week 5 and week 10 and a linear increase (*p* < .05) in serum total protein concentration on week 10 of pigs fed diets supplemented with graded levels of AJE was observed. Faecal lactic acid bacteria counts showed a linear increase (*p* < .05) on week 5 with the increasing levels of AJE.

**Conclusions:**

In conclusion, there existed improvements in growth performance, nutrients digestibility, serum total protein, faecal coliform bacteria and lactic acid bacteria counts and faecal ammonia emission in the finishing pigs fed with AJE‐supplemented diet.

## Abstract 2

This study was evaluated the effects of dietary supplementation of *Achyranthes japonica* extracts (AJE) on the performance and production parameters in finishing pigs. In conclusion, there existed improvements in growth performance, nutrients digestibility, serum total protein, faecal coliform bacteria and lactic acid bacteria counts and faecal ammonia emission in the finishing pigs fed with AJE‐supplemented diet.

## 1 INTRODUCTION

With the ban of antibiotics for the health and growth of animals, there has been considerable research interest in developing alternatives to antibiotic growth promoters (Levy, 2014). *Achyranthes japonica* belongs to the genus of *Achyranthes* in the family of *Amaranthaceae*. The root contains multiple active constituents, such as phytoecdysteroid, saponin, polysaccharide, inokosterone and 20‐hydroxyecdysone (Park et al., 2013). It is mainly distributed in China, Korea and Japan. In South Korea, *Achyranthes japonica* extracts (AJE) is used to be a traditional medicine as an analgesic and diuretic to treat rheumatism, osteoarthritis and hypertension (Jung et al., 2007). Previously, it was observed that *Achyranthes japonica* extracts (AJE) contained various positive physiological benefits, such as anti‐allergic, hepato‐protective, anti‐inflammatory, alleviation, antioxidant, anti‐cancer and arthritis functions (Bang et al., 2012; Jung et al., 2007; Kim & park, 2010). Previous studies were indicated that dietary addition with AJE improved the growth performance in broiler chickens (Park & Kim, 2020; Sun et al., 2020). In addition, Liu et al., (2020) suggested that dietary supplementation with AJE enhances the growth performance and nutrient digestibility in growing pigs.

Considering the above benefits, it is hypothesised that the AJE might have positive effects on the performance of finishing pigs. However, to the best of our knowledge, the effects of dietary supplementation with AJE in pigs’ diets remain largely unknown. Therefore, the aim of this study was to determine the effects of AJE supplementation on the growth performance, nutrient digestibility, blood characteristics, faecal microbial shedding, faecal noxious gas emission and meat quality in finishing pigs.

## 2 MATERIALS AND METHODS

### 2.1 Product preparation

The AJE used in this study was a commercial product manufactured by a company (Synergen Inc., Bucheon, Korea). The *Achyranthes japonica* was cultivated in South Korea. After wash, the roots of *Achyranthes japonica* were powdered with a mill (IKAM20; IKA, Staufen, Germany). The samples were extracted with distilled water at 80°C and then refluxed for 6 hr to obtain the initial extract. The residues were extracted with distilled water (1:5) at 80°C for 2 hr, and the extract solution was filtered under low temperature by a high‐velocity centrifugal machine. Useful parts were collected in a column and eluted with ethanol. The samples were vacuum‐dried under 40°C after cooling and filtering. The final extracts were completely dried by a freeze drier. The AJE contains active constituents of total flavonoid (1.15 mg/g), total polyphenol (4.26 mg/g) and saponin (0.47 mg/g).

### 2.2 Experimental design, animals and diets

Totally, 100 cross‐breed finishing pigs [(Landrace × Yorkshire) × Duroc] with the average initial body weight (BW) of 50.33 ± 4.61 kg were used in this 10 weeks feeding experiment. Pigs were allotted to four dietary treatments in a randomised complete block design according to their BW (five replicates, three barrows and two gilts per pen), containing the addition of 0%, 0.05%, 0.10% and 0.20% AJE. All the diets were formulated to meet or exceed the NRC (2012) (Table 1). To allow ad libitum access to feed and water throughout the experiment, each pen was fitted with a stainless steel feeder and one nipple drinker.

**TABLE 1:** Ingredient composition of experimental diets as fed basis

Items	
Ingredients, %	
Corn	79.21
Soybean meal	16.20
Tallow	1.72
Tribasic calcium phosphate	1.18
Limestone	0.41
Salt	0.20
Methionine (99%)	0.06
Lysine	0.44
Threonine (99%)	0.13
Tryptophan (10%)	0.20
Mineral mix^†^	0.10
Vitamin mix^‡^	0.12
Choline (25%)	0.03
Calculated composition	
Crude protein, %	14.50
Calcium, %	0.61
Phosphorus, %	0.54
Lysine, %	1.00
Methionine, %	0.29
Threonine, %	0.66
Tryptophan, %	0.18
Crude fat, %	4.69
Crude fibre, %	2.43
Ash, %	3.85
Metabolisable energy (kcal/kg)	3,300

^†^ Provided per kg of complete diet: 11,025 IU vitamin A; 1,103 IU vitamin D_3_; 44 IU vitamin E; 4.4 mg vitamin K; 8.3 mg riboflavin; 50 mg niacin; 4 mg thiamine; 29 mg d‐pantothenic; 166 mg choline; 33 μg vitamin B_12_.

^‡^ Provided per kg of complete diet: 12 mg Cu (as CuSO_4_.5H_2_O); 85 mg Zn (as ZnSO_4_); 8 mg Mn (as MnO_2_); 0.28 mg I (as KI); 0.15 mg Se (as Na_2_SeO_3_.5H_2_O).

### 2.3 Sampling and measurements

The pigs were weighed at the beginning, week 5 and week 10, and then weekly feed consumption was recorded on a pen basis during the experiment period. The average daily gain (ADG), feed conversion ratio (FCR) and average daily feed intake (ADFI) were calculated.

During week 5 and week 10, the apparent total tract digestibility (ATTD) of dry matter (DM), crude protein (CP) and fat were evaluated by adding 0.2% chromic oxide (Cr_2_O_3_) to the diets as an indigestible marker. Two pigs from each pen were randomly chosen to take the fresh faecal during the last 3 days on week 5 and week 10. All the fresh samples were put in a freezer at −20°C for further analysis. Before chemical analysis, the faecal samples need to be thawed and dried at 70°C for 72 hr and then ground to fit through a 1 mm sieve. All of the feed and faecal samples were then analysed for DM, CP and fat following the procedures outlined by the AOAC (2005). Chromium was analysed using a machine (Shimadzu, UV‐1201, Kyoto, Japan) and crdue protein was determined using a Kjeltec 2,300 Analyser machine (Foss Tecator AB, Hoeganaes, Sweden). Using the following formula: $\text{digestibility}\left(\%\right)=\left\{1‐\left[\left(\text{Nf}\times \text{Cd}\right)/\left(\text{Nd}\times \text{Cf}\right)\right]\right\}\times 100$, where, Nf =nutrient concentration in faeces (% DM), Nd = nutrient concentration in diet (% DM), Cd =chromium concentration in diet (% DM) and Cf = chromium concentration in faeces (% DM).

At the last day of week 5 and week 10, two pigs per pen were randomly selected for collecting 5 ml fresh blood samples by the jugular venipuncture into vacuum tubes (Becton Dickinson Vacutainer Systems, Franklin Lakes, NJ, USA) and centrifuged at 3,000 × g for 20 min at 4°C. The glucose, total protein, blood urea nitrogen (BUN) and creatinine were analysed by an automatic blood analyzer (HITACHI 747, Japan).

Fresh faecal samples were collected from two pigs each pen by anal massage and then directly put into micro‐tubes at the last day of week 5 and week 10. One gram of the faecal sample was diluted with 9 ml of 1% peptone broth (Becton, Dickinson and Co., Franklin Lakes, NJ) and then homogenised. Coliform bacteria and lactic acid bacteria were isolated by inoculation of MacConkey agar plates (Difco Laboratories, Detroit, MI) and Lactobacillus medium agar plates (Medium 638, DSMZ, Braunschweig, Germany) with 10 times continuous diluent (1% peptone solution). The Lactobacilli medium agar plates and the MacConkey agar plates were then incubated for 48 hr at 39°C and 24 hr at 37°C under anaerobic conditions, respectively. The lactic acid bacteria and coliform bacteria were counted immediately after being removed from the incubator.

Faeces were collected by rectum massage to determine faecal NH_3_, hydrogen sulfide (H_2_S) and total mercaptans emission on week 5 and week 10. Fresh faeces (150 g) were stored in a 2.6 L plastic box for the replicates (Yan et al., 2011). The samples were fermented for 7 days at room temperature. A gas sampling pump was used for gas detection. The adhesive plaster was punctured and 100 ml of headspace air was collected about 2 cm above the faecal surface for 1 min.

The pigs were transported to a local commercial slaughterhouse and then slaughtered immediately. After cooling for 24 hr at 2°C, a piece of the right loin sample was removed between the 10th and 11th ribs. Following the National Pork Producers Council Standards, sensory evaluation (colour, firmness and marbling scores) was conducted at ambient temperature. Immediately after the subjective tests were conducted, the lightness (L*), yellowness (b*) and redness (a*) values were measured at three locations on the surface of each sample using a machine (Model CR‐410 Chroma metre, Konica Minolta Sensin Inc., Osaka, Japan). After that, duplicate pH values of every sample were measured by a pH meter (Pittsburgh, PA, USA). The water holding capacity (WHC) and drip loss were measured according to the methods described by Kauffman et al. (1986) and Honikel (1998), respectively. The water holding capacity (WHC) was determined by a digitising area‐line sensor (MT‐10S; M.T. Precision Co. Ltd., Tokyo, Japan). Four grams of meat sample was heated to 70°C in a water bath for 30 min. Cooking loss was calculated as the weight difference between the initial raw and final cooked samples. Longissimus muscle (LM) area was measured by a digitising area‐line sensor (MT‐10S; M.T. Precision Co. Ltd., Tokyo, Japan) to trace the LM surface at the 10th rib. Backfat thickness and lean meat percentage were measured at week 5 and week 10. Lean meat percentage was measured by Pig‐log 105 (Carometec Food Technology, Denmark) at 6.5 cm area on the right and left end frames. Lean meat percentage was calculated according to NPPC (1999) procedures with a proprietary equation.

### 2.4 Statistical analysis

The pen was considered as the experimental unit, and all data were analysed with SAS (SAS Inst. Inc., Cary, NC) by using mixed procedures. Differences between groups were used Duncan's multiple range tests. Orthogonal polynomials were used to evaluate the linear and quadratic effects. A *p* < .05 was considered as significant, and 0.05 < *p* < .10 was considered as trends.

### 2.5 Animal care

The management and care of animal experimental protocols described in this research were assessed and accepted by the Dankook University Use Committee and Animal Care (Case No. DK‐4–1628), Republic of Korea.

## 3 RESULTS

### 3.1 Growth performance

Following the increasing levels of AJE addition, the ADG of pigs were linearly increased (*p* < .05) during week 5, week 10 and the overall experimental period. Furthermore, during week 5, the FCR was linearly decreased (*p* < .05) with the increasing dosage of AJE supplementation. However, no significant effects (*p* > .10) were observed on ADFI during all the experimental periods (Table 2).

**TABLE 2:** Effect of dietary AJE supplementation on growth performance in finishing pigs

Items	CON	TRT1	TRT2	TRT3	*SEM* ^†^	*p*‐value	
						Linear	Quadratic
Week 1–5							
ADG, g	790^a,b^	802^a,b^	819^a,b^	832^a,b^	11	0.0111	0.9414
ADFI, g	2,398	2,400	2,431	2,383	23	0.8939	0.2968
FCR	3.035^a,b^	2.993^a,b^	2.969^a,b^	2.865^a,b^	0.045	0.0240	0.5557
Week 6–10							
ADG, g	853^a,b^	862^a,b^	867^a,b^	891^a,b^	11	0.0347	0.5109
ADFI, g	2,995	3,025	3,027	3,054	30	0.2156	0.9588
FCR	3.512	3.510	3.491	3.427	0.067	0.4316	0.6819
Overall							
ADG, g	821^a,b^	832^a,b^	843^a,b^	862^a,b^	10	0.0128	0.6773
ADFI, g	2,696	2,713	2,729	2,718	20	0.3651	0.5024
FCR	3.285	3.264	3.242	3.166	0.051	0.1232	0.5956

Abbreviation: AJE, *Achyranthes japonica* extracts; ADG, average daily gain; ADFI, average daily feed intake; FCR, feed conversion ratio; CON, Basal diet; TRT1, Basal diet +0.05% AJE; TRT2, Basal diet + 0.10% AJE; TRT3, Basal diet + 0.20% AJE.

^†^ Standard error of means.

^a,b^ Means in the same row with different superscripts differ significantly (*p* < .05).

### 3.2 Nutrient digestibility

As shown in Table 3, linear increases (*p* < .05) of DM and CP digestibility were observed at week 5 and week 10 with dietary increasing levels of AJE supplementation. However, no significant effects (*p* > .10) were shown on the fat digestibility at both week 5 and week 10.

**TABLE 3:** Effect of dietary AJE supplementation on nutrient digestibility in finishing pigs

Items, %	CON	TRT1	TRT2	TRT3	*SEM* ^†^	*p*‐value	
						Linear	Quadratic
Week 5							
Dry matter	73.54^a,b^	74.64^a,b^	75.07^a,b^	76.48^a,b^	0.61	0.0026	0.8005
Crude Protein	72.61^a,b^	74.38^a,b^	75.12^a,b^	76.41^a,b^	0.94	0.0087	0.7991
Fat	65.18	66.19	65.79	66.21	0.94	0.5253	0.7562
Week 10							
Dry matter	69.92^a,b^	71.51^a,b^	71.66^a,b^	72.64^a,b^	0.78	0.0266	0.6999
Crude Protein	68.90^a,b^	71.23^a,b^	71.75^a,b^	72.55^a,b^	1.12	0.0331	0.5135
Fat	60.80	61.52	61.37	61.98	1.18	0.5265	0.9632

Abbreviation: AJE, *Achyranthes japonica* extracts; CON, Basal diet; TRT1, Basal diet + 0.05% AJE; TRT2, Basal diet + 0.10% AJE; TRT3, Basal diet + 0.20% AJE.

^†^ Standard error of means.

^a,b^ Means in the same row with different superscripts differ significantly (*p* < .05).

### 3.3 Blood characteristics

No significant effects (*p* > .10) were observed on glucose, BUN and creatinine concentration of pigs fed AJE‐supplemented diets. However, there was a linear increase (*p* < .05) in serum total protein concentration of pigs fed diets supplemented with graded levels of AJE on week 10 (Table 4).

**TABLE 4:** Effect of dietary AJE supplementation on blood profile in finishing pigs

Items, mg/dl	CON	TRT1	TRT2	TRT3	*SEM* ^†^	*p*‐value	
						Linear	Quadratic
Week 5							
Glucose	90.0	92.3	89.5	90.3	4.2	0.9174	0.8620
Total protein, g/dl	7.0	7.1	7.3	7.4	0.2	0.1309	0.8676
Blood urea nitrogen	6.8	6.5	6.0	6.0	0.6	0.3426	0.8432
Creatinine	1.1	1.2	1.2	1.1	0.1	0.7082	0.0182
Week 10							
Glucose	94.5	91.8	94.0	93.8	4.4	1.0000	0.7848
Total protein, g/dl	8.0^a,b^	8.2^a,b^	8.3^a,b^	8.6^a,b^	0.1	0.0138	0.5625
Blood urea nitrogen	10.0	9.8	9.5	9.3	0.6	0.3534	1.0000
Creatinine	1.4	1.5	1.5	1.3	0.1	0.6368	0.0828

Abbreviation: AJE, *Achyranthes japonica* extracts; CON, Basal diet; TRT1, Basal diet + 0.05% AJE; TRT2, Basal diet + 0.10% AJE; TRT3, Basal diet + 0.20% AJE.

^†^ Standard error of means.

^a,b^ Means in the same row with different superscripts differ significantly (*p* < .05).

### 3.4 Faecal microbial shedding

As described in Table 5, faecal lactic acid bacteria counts showed a linear increase (*p* < .05) on week 5, and faecal coliform bacteria counts showed a linear decrease tendency (0.05 < *p* <.10) on both week 5 and week 10 with the increased dietary supplementation of the AJE.

**TABLE 5:** Effect of dietary AJE supplementation on faecal microbial shedding in finishing pigs

Items, log_10 cfu_/g	CON	TRT1	TRT2	TRT3	*SEM* ^†^	*p*‐value	
						Linear	Quadratic
Week 5							
Lactic acid bacteria	7.31^a,b^	7.34^a,b^	7.39^a,b^	7.41^a,b^	0.03	0.0309	0.8347
Coliform bacteria	5.80	5.77	5.68	5.65	0.06	0.0891	1.0000
Week 10							
Lactic acid bacteria	7.70	7.73	7.69	7.72	0.03	0.8785	0.9319
Coliform bacteria	5.89	5.88	5.80	5.74	0.06	0.0594	0.6441

Abbreviation: AJE, *Achyranthes japonica* extracts; CON, Basal diet; TRT1, Basal diet + 0.05% AJE; TRT2, Basal diet + 0.10% AJE; TRT3, Basal diet + 0.20% AJE.

^†^ Standard error of means.

^a,b^ Means in the same row with different superscripts differ significantly (*p* < .05).

### 3.5 Faecal gas emission

On week 5 and week 10, there was a linear decline (*p* < .05) in faecal NH_3_ emission followed by the dietary AJE supplementation from 0% to 0.20%, whereas no differences (*p* > .10) were observed in faecal H_2_S and total mercaptans emission (Table 6).

**TABLE 6:** Effect of dietary AJE supplementation on faecal gas emission in finishing pigs

Items, ppm	CON	TRT1	TRT2	TRT3	*SEM* ^†^	*p*‐value	
						Linear	Quadratic
Week 5							
Ammonia	6.44^a,b^	5.95^a,b^	5.83^a,b^	5.51^a,b^	0.25	0.0235	0.7529
Total mercaptans	2.99	2.76	2.74	2.60	0.25	0.2915	0.9054
Hydrogen sulfide	3.09	3.05	2.96	2.85	0.32	0.6192	0.9278
Week 10							
Ammonia	6.84^a,b^	6.64^a,b^	6.58^a,b^	5.80^a,b^	0.28	0.0280	0.3285
Total mercaptans	3.26	3.12	2.84	2.64	0.44	0.2983	0.9468
Hydrogen sulfide	3.54	3.32	3.30	3.12	0.30	0.3581	0.9479

Abbreviation: AJE, *Achyranthes japonica* extracts; CON, Basal diet; TRT1, Basal diet + 0.05% AJE; TRT2, Basal diet + 0.10% AJE; TRT3, Basal diet + 0.20% AJE.

^†^ Standard error of means.

^a,b^ Means in the same row with different superscripts differ significantly (*p* < .05).

### 3.6 Meat quality

Meat quality was unaffected by dietary AJE supplementation throughout the experiment (*p* > .10; Table 7).

**TABLE 7:** Effect of dietary AJE supplementation on meat quality in finishing pigs

Items	CON	TRT1	TRT2	TRT3	*SEM* ^†^	*p*‐value	
						Linear	Quadratic
Meat colour							
L*	47.19	48.11	47.24	47.98	0.38	0.3949	0.8259
a*	15.54	15.20	14.93	14.96	0.35	0.2287	0.6141
b*	3.41	3.25	3.84	3.67	0.39	0.4537	0.9950
Sensory evaluation							
Color	3.34	3.41	3.38	3.44	0.07	0.4414	1.0000
Firmness	2.44	2.44	2.50	2.50	0.10	0.3556	0.4788
Marbling	2.25	2.28	2.38	2.31	0.06	0.5885	0.9904
Cooking loss, %	38.10	34.15	37.83	34.52	1.66	0.3668	0.8524
Drip loss,%							
d1	7.09	7.15	6.93	7.38	1.31	0.9146	0.8844
d3	13.09	13.53	13.11	12.94	1.26	0.8795	0.8146
d5	19.84	19.4	19.67	18.68	0.65	0.3029	0.6855
d7	22.96	22.96	22.67	22.87	0.34	0.7110	0.7748
pH	5.49	5.38	5.42	5.51	0.10	0.8398	0.3398
Water holding capacity, %	47.92	48.11	49.31	51.42	2.33	0.2918	0.6912
Longissimus muscle area, cm^2^	59.96	60.36	62.00	62.93	1.48	0.1460	0.8608

Abbreviation: AJE, *Achyranthes japonica* extracts; CON, Basal diet; TRT1, Basal diet + 0.05% AJE; TRT2, Basal diet + 0.10% AJE; TRT3, Basal diet + 0.20% AJE.

^†^ Standard error of means.

## 4 DISCUSSION

With the increasing restriction for livestock farm managers to decrease the usage of antibiotics, medicinal herb has been the issue of many types of research as a potential replacement for antibiotics in the livestock industry (Hernandez et al., 2004). In the present study, an improvement in ADG and FCR was obtained in pigs fed AJE‐containing diets. In agreement with this research, Liu et al. (2020) showed that dietary supplementation with AJE led to a greater ADG and gain to feed ratio than those fed with non‐supplementation diets in growing pigs. Conversely, O’Sullivan et al., (2003) reported that the dietary addition of herbal extract (citrus pulp) had no effects on the growth performance in growing pigs. The inconsistent results might because of the different herb species, the dose of herb extract and the different growth phase of experimental animals. Currently, despite there were not enough researches related to the use of AJE with pigs, and also the mode of action of AJE on the growth performance of pigs has not been clearly understood. According to our previous study, Park and Kim (2020) have been suggested that dietary addition with an increasing level of AJE linearly improved the broilers’ growth performance. Similarly, Liu et al. (2018) demonstrated that the ADG and final BW of Yellow Broiler Chickens were increased significantly with the *Achyranthes* plants extract treatment compared to the control group. Although the experimental animals in the above studies are broilers, and the physiological structures of broilers and pigs are different to some extent, the experimental results on broilers are consistent with the results on pigs in this study. Since AJE contains a wide range of bioactive substances, such as saponin, polyphenol compounds, these lead to a variety of positive physiological effects such as antioxidative properties, antimicro‐bioactivity and immunostimulating effect (Liu et al., 2008). These positive physiological effects may lead to an improvement in immune response and intestinal flora balance and an increase in digestibility and nutrient absorption. Therefore, it is likely that the improvement in the growth performance of pigs fed AJE diets in this study may be due to these bioactive phytochemicals and their positive physiological effects. The current research showed that the ATTD of CP and DM was significantly increased linearly followed by the increasing levels of dietary AJE inclusion during both week 5 and week 10 in finishing pigs, which indicated that the greater digestibility could be achieved with the addition of AJE in diet. In agree with this study, Liu et al. (2020) indicated that dietary supplementation with AJE increased the ATTD of DM in growing pigs. Sun et al. (2020) showed that dietary supplementation with AJE improved the ATTD of nitrogen and DM in broiler chickens. Similarly, Park and Kim (2020) indicated that dietary inclusion of increasing AJE linearly improved the ATTD of DM and nitrogen in broilers. These consistent results may be due to the fact that herbal extracts have positive active substances that promote digestion and nutrient metabolism, thereby stimulating the growth performance of pigs (Czech et al., 2009). Additionally, previous studies showed that supplementation with phytogenic feed additives increased villus length and decreased crypt depth, which is indicated an improvement in nutrient absorption (Namkung et al., 2004; Oetting et al., 2006). It is generally accepted that herb extracts could stimulate the secretion of mucus by the stomach and intestine, thereby preventing pathogens from attaching, helping to stabilise the beneficial flora and protecting the intestinal villi, improving absorption and digestion of nutrients (Costa et al., 2013). Therefore, the increasing digestive capacity and the stable balance of intestinal microorganisms by AJE stimulation may be the main reasons for the increased nutrients digestibility of pigs.

In this study, the dietary supplementation of AJE showed linearly increased the total serum protein concentration but did not affect the glucose concentration. The studies about the effects of herb extracts on total serum protein in swine were limited. However, the results of the feeding experiment indicated that the addition of AJE to the pig diets increased the crude protein digestibility. The increased crude protein digestibility, which promotes the synthesis of serum total protein, may be one reason for the increase in serum total protein concentration (Tang et al., 2005). Serum total protein concentration may also reflect the status of hepatic protein metabolism. Therefore, the increased serum total protein content demonstrates that the addition of AJE to diet may improve the hepatic function of pigs to some extent in pigs. Interestingly, results suggested that the AJE have a beneficial effect on the gut microbial balance; it is observed that the dietary inclusion of AJE supplementation decreased the faecal coliform bacteria counts and increased lactic acid bacteria counts in the present study. Also, Liu et al. (2020) demonstrated that dietary addition of AJE reduced the faecal coliform bacteria counts and increased lactic acid bacteria counts in growing pigs. Park and Kim (2020) reported that broilers fed an AJE supplementation diet decreased *E*. *coli* counts and increased *Lactobacillus* count. According to the study of Xie et al. (2018) reported that the addition of *Achyranthes bidentata* extract increased the growth of *Lactobacillus*. It is fully known that herbs have antibacterial effects against pathogens in vitro (Si et al., 2006). Similarly in an in vivo study, Windisch et al. (2008) reported that herbs could increase the resistance of animals under different stress conditions by inhibiting the growth of pathogenic microorganisms in the gastrointestinal ecosystem. Chen et al. (2009) demonstrated that the *Achyranthes bidentata* extract in addition to the diets reduced the diarrhoea frequency of weaning pigs, indicating the inhibitory effect on enteric pathogens. The mechanism of antibacterial action of herb extracts is by acting on bacterial cell wall structures denatured and coagulated proteins (Costa et al., 2013). Therefore, the antibacterial effect of AJE may be the main cause of the decrease in coliform bacteria counts in this study. The lower coliform bacteria counts lead to a balance of intestinal flora that promotes intestinal health status, which in turn helps increase the counts of favourable microbial community such as lactic acid bacteria. In addition, the balance of intestinal microbial flora may also be one of the reasons for better nutrient absorption, thereby improving digestibility and increasing the growth performance in pigs. Ammonia is the main component of odour emission from pig farms and it not only contributes to environment pollution but also causes irritation in the respiratory system of pigs and humans (Sun et al., 2008). In this research, the dietary supplementation of AJE into diets linearly decreased the faecal NH_3_ emission. Similar to the results of this study, Yan et al. (2010) observed a significant reduction of NH_3_ in pigs fed thyme, rosemary and oreganum extract essential oil in finishing pigs. Moreover, Sun et al. (2020) indicated that the supplementation of AJE reduced the faecal NH_3_ emission in broilers. Recently, Park and Kim (2020) indicated that the addition of AJE decreased the excreta emission of NH_3_ in broilers. It was suggested that the faecal noxious gas emission of pigs is related to the intestinal harmful bacteria population (Ferket et al., 2002). In this experiment, the increased faecal *Lactobacillus* counts could consider as an improvement in microflora balance in pigs which may prevent the colonisation of harmful bacteria, consequently reduce the faecal gas emission (Lan et al., 2016). Additionally, it was reported that the NH_3_ as the major nitrogenous gas emission in pig farms is mainly produced by the microbial decomposition of the undigested dietary nitrogen (Jha and Berrocoso, 2016). Thus, the increased digestibility of protein is considered as the other reason for reducing faecal NH_3_ emission. The improvement in digestibility resulted in less substrate for the microbial fermentation, thereby decreases the NH_3_ emission (Yan et al., 2011a).

In the present research, the analysed meat quality parameters, such as meat colour, sensory evaluation, cooking loss percentage, drip loss percentage, pH, WHC and LM area percentage, were not affected by dietary supplementation of AJE. The influences of herbal extracts have been reported in improvement in meat quality of pork. For instance, Yan et al. (2011b) indicated that dietary supplementation of *Taraxacum officinale* and *Houttuynia cordata* extracts increased the LM area and 2‐Thiobarbituric acid reactive substances value in finishing pigs. However, the exact mechanism of herbal extracts influencing the meat quality was not fully understood. In line with the current research, Ao et al. (2011) reported that addition of fermented red ginseng did not influence the meat quality parameters in finishing pigs. Recently, Liu et al. (2016) investigated that *Lonicera japonica* *and* *Scutellaria baicalensis* extracts mixture supplementation did not affect meat colour, drip loss, sensory evaluation and cooking loss in finishing pigs. There have many factors that could impact the meat quality parameters. The inconsistent results could be due to the different diet and species and dosage of herbal extracts inclusion. Moreover, the unaffected meat quality parameters also could be concluded as no deleterious effects on consumer acceptability with dietary supplementation of AJE. This conclusion would be worthy for further studies.

## 5 CONCLUSION

In summary, dietary inclusion of increasing levels of AJE improved the growth performance and nutrients digestibility, increased the serum total protein concentration, balanced the gut microbial and reduced the faecal NH_3_ emission in finishing pigs. The results of this study provide a positive basis for further research on the application of herbal extracts in finishing pigs.

## CONFLICT OF INTEREST

We confirm that there are no conflicts of interest associated with this publication.

## AUTHOR CONTRIBUTION

**Xiao Liu:** Conceptualization; Data curation; Formal analysis; Investigation; Methodology; Writing‐original draft; Writing‐review & editing. **In Ho Kim:** Funding acquisition; Project administration; Writing‐review & editing.

## Data Availability

The data used to support the findings of this study are available from the corresponding author upon request.
